# Pet-directed speech improves horses’ attention toward humans

**DOI:** 10.1038/s41598-022-08109-z

**Published:** 2022-03-11

**Authors:** Plotine Jardat, Ludovic Calandreau, Vitor Ferreira, Chloé Gouyet, Céline Parias, Fabrice Reigner, Léa Lansade

**Affiliations:** 1 CNRS, IFCE, INRAE, University of Tours, PRC, 37380 Nouzilly, France; 2 Department of Physics, Chemistry and Biology, IFM Biology, Linköping University, 581 83 15 Linköping, Sweden; 3 UEPAO, INRAE, 37380 Nouzilly, France

**Keywords:** Ecology, Neuroscience, Ecology

## Abstract

In a recent experiment, we showed that horses are sensitive to pet-directed speech (PDS), a kind of speech used to talk to companion animals that is characterized by high pitch and wide pitch variations. When talked to in PDS rather than adult-directed speech (ADS), horses reacted more favorably during grooming and in a pointing task. However, the mechanism behind their response remains unclear: does PDS draw horses’ attention and arouse them, or does it make their emotional state more positive? In this study, we used an innovative paradigm in which female horses watched videos of humans speaking in PDS or ADS to better understand this phenomenon. Horses reacted differently to the videos of PDS and ADS: they were significantly more attentive and their heart rates increased significantly more during PDS than during ADS. We found no difference in the expressions of negative or positive emotional states during PDS and ADS videos. Thus, we confirm that horses’ perception of humans can be studied by means of video projections, and we conclude that PDS attracts attention and has an arousing effect in horses, with consequences on the use of PDS in daily interactions with them.

## Introduction

In the past 20 years, an increasing number of studies have explored the cognitive abilities of domestic mammals toward humans, bringing to light sometimes unexpected sociocognitive skills^1^. For example, sheep recognize individual human faces^2^, goats know when humans are attentive to them^3^, and dogs and cats recognize and react to our emotions^4–6^. These findings enable us to improve human-animal interactions and animal welfare, as we better understand how our actions and emotions affect domestic mammals. Horses also have excellent sociocognitive skills toward humans. They recognize our emotions through vocalizations, facial expressions and odors^7–11^, they know whether we are attentive to them^12^ and what the intentions of our gestures are^13^. This may be linked to proximity to humans since their domestication approximately 5500 years ago^14^ and to the importance of social interactions in this species^15^.

Horses are also sensitive to the way we talk to them, especially to a kind of speech used to speak to companion animals, called pet-directed speech (PDS). PDS resembles the type of speech used with infants (“baby-talk”, “parentese” or “infant-directed speech”—IDS)^16^ and has similar characteristics, namely, a high and varying pitch, wide pitch range, slow rate of speech, simple syntax and semantics and more repeated words, compared to adult-directed speech (ADS)^16–21^. Moreover, IDS is an emotional form of speech and a multimodal communication style^16^, which includes visual modalities such as facial expressions, particularly smiles. IDS and PDS effects have been extensively studied in human infants and other animal species, such as primates and dogs. In human infants, IDS is preferred to ADS^22^; it has an arousing effect and facilitates social interactions^16^. Infants’ emotions are influenced by IDS, with 5-month-old infants smiling more in response to approval expressed in IDS than ADS^23^. In infant rhesus macaques, IDS has been shown to influence long-term memory^24^. Moreover, dogs looked longer at and preferred to spend time close to a loudspeaker or person broadcasting PDS rather than ADS^17,18^, and they were more attentive when spoken to in PDS rather than ADS^19^. In horses, we showed in a recent experiment that PDS induced a different way of interacting with an experimenter compared to ADS. While being scratched on the withers, horses moved less, made more grooming attempts (i.e., mouth gestures equivalent to those made when grooming a conspecific), and looked more at the experimenter when they talked in PDS rather than ADS. Furthermore, in a pointing task in which the horses had to find the location of a food reward from cues given by the experimenter, horses that were talked to in PDS performed better than horses that were talked to in ADS^21^.

In connection with the observations made in other species, we proposed several hypotheses to explain this reaction of horses to PDS. First, PDS could attract horses’ attention and arouse them. Indeed, IDS is arousing for infants^16,25^, and PDS attracts attention in dogs^17–19^. This hypothesis could explain why horses looked more at the experimenter during grooming and their better performance in finding hidden food due to greater attention given to experimenter cues. Second, PDS could influence the valence of emotions felt by horses. Indeed, the positive emotions expressed through PDS could make horses react positively to this type of speech. In fact, horses have been observed to relax in response to joyful intonations of humans^10,11^, and PDS could have similar consequences, in the same way that IDS is known to trigger positive emotions in infants^16,23^. Moreover, in dogs and infants, the preference for IDS or PDS over ADS also suggests a more positive emotional experience in response to the former. Thus, in our first study on PDS in horses, a more positive emotional state, linked to the positive emotional charge of PDS, could have lead horses to make more grooming attempts (as in^26^). In addition, it could have helped them follow the experimenter’s indications when pointing toward the food reward due to the better cognitive performance that results from a more positive emotional state^27,28^.

The aim of this study was to determine whether horses would have different behavioral and physiological reactions when presented with films of humans speaking in PDS or ADS and to explore two nonexclusive hypotheses, **H1** and **H2,** that can explain horses’ sensitivity to PDS:

**H1**: PDS helps to attract horses’ attention and has an arousing effect on them.

**H2**: PDS influences the valence of emotions felt by horses, making them more positive.

We used an innovative paradigm in which horses watched videos of humans that had been previously filmed in the laboratory. Horses are known to react to projected films of humans or conspecifics, with an influence on their emotional state and behavior^11,29^. We showed 28 horses two-minute-long films composed of four 30-s sections, each consisting of a woman saying the same sentence four times in either PDS or ADS. To test our two hypotheses, the propensity to be more attentive to PDS than ADS, relative to the total time spent being attentive to the screen (hereafter called the attention index) was registered, and behavioral indicators of emotional valence were observed. The time spent in an alert posture and signs of fear (number of defecations, number of times the white of the eye was visible—called shows of sclera, number of neighs)^30,31^, which reflect a negative emotional state, were recorded. The time spent in a relaxed posture and the number of snorts^32^, which reflect a positive emotional state, were also quantified. The horses’ heart rates were also recorded as a possible marker of either emotional valence^8,11^ or arousal^33,34^ (the two axes of the dimensional approach of emotional states in animals^35^, where valence describes the variation between positive and negative experiences and arousal is defined as the degree of “bodily activation, e.g. calm versus excited”^33,35^).

According to **H1**, we expected horses to be attentive for a longer period of time during PDS than during ADS, with a possible greater increase in heart rate during PDS due to increased arousal. According to **H2**, we expected horses to show more signs of a positive emotional state (relaxed posture, snorts) during PDS than during ADS, with a possible greater decrease in heart rate and fewer behavioral signs of a negative emotional state (e.g., alert postures, neighs, defecations, shows of sclera) during the former.

## Results

**Figure 1:**
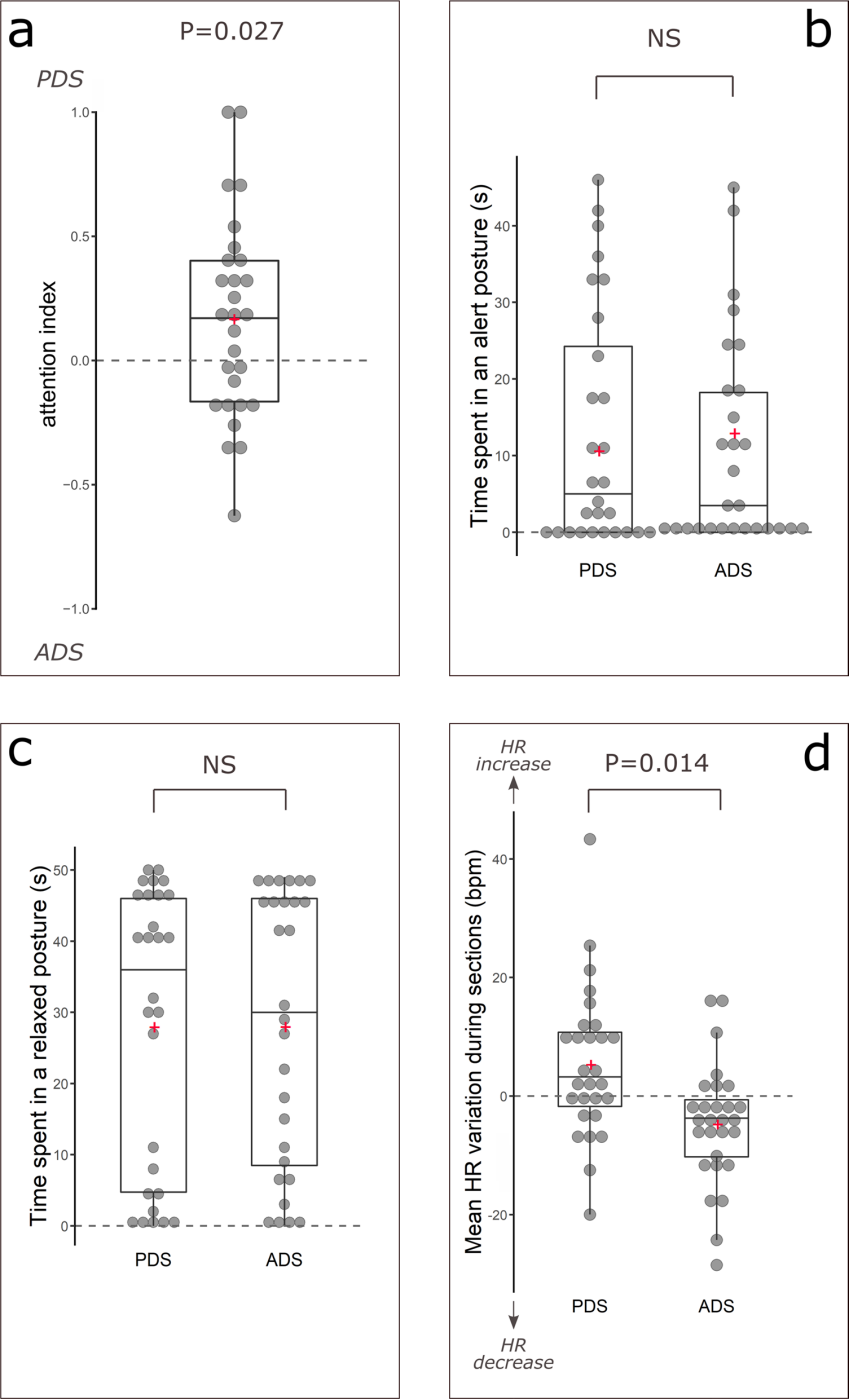
Horses’ behavior and heart rate variations during PDS and ADS. Boxplots showing median, first and third quartiles;. Grey dot: individual results (N = 28); Red Plus symbol: mean. Wilcoxon tests, *NS* not significant. (**a**) Attention index: (A^PDS^-A^ADS^)/(A^PDS^ + A^ADS^) with A^PDS^ the time spent being attentive to the screen during PDS sections, and A^ADS^ during ADS sections. (**b**) Time spent in an alert posture when ADS or PDS was projected (s). (**c**) Time spent in a relaxed posture when ADS or PDS was projected (s). (**d**) Mean difference in heart rate between the last five and the first five seconds of the sections when ADS or PDS was projected (bpm).

The attention index (measuring the propensity to be more attentive to PDS than ADS) was significantly greater than 0 (one-tailed Wilcoxon test, N = 28, V = 288, P = 0.027, Fig. 1a), indicating that the horses were more attentive to the videos when the human was speaking in PDS rather than ADS, relative to the total time spent being attentive to the screen.

The time spent in an alert posture was not lower during PDS sections than during ADS sections (one-tailed paired Wilcoxon test, N = 28, U = 61, P = 0.952; Fig. 1b), and the time spent in a relaxed posture was not higher during PDS sections than during ADS sections (one-tailed paired Wilcoxon test, N = 28, U = 175, P = 0.632; Fig. 1c). The proportions of horses that defecated, neighed and showed the sclera at least once during PDS were not lower than those during ADS (one-tailed Z tests, P = 28, defecations: PDS 1/28, ADS 2/28, Z = 0.593, P = 0.500; neighs: PDS 2/28, ADS 4/28, Z = 0.864, P = 0.333; shows of sclera: PDS 5/28, ADS 7/28, Z = 0.651, P = 0.372). Furthermore, we did not observe any snorts during the tests.

The mean difference in heart rate between the end and the beginning of the sections was significantly higher for PDS than ADS (two-tailed paired Wilcoxon test, N = 28, V = 96, P = 0.014, Fig. 1d), and this variation was different from 0 during both PDS and ADS sections (two-tailed Wilcoxon tests, N = 28; PDS: V = 294, P = 0.038; ADS: V = 87, P = 0.007), indicating that horses’ heart rate increased during the PDS film sections and decreased during the ADS film sections.

## Discussion

In this experiment, horses had different behavioral and physiological reactions when presented with films of humans speaking in PDS or ADS. Horses were more attentive to PDS videos than ADS videos, and their heart rates increased more during PDS than during ADS; however, they did not show fewer signs of a negative emotional state or more signs of a positive emotional state during PDS than during ADS.

In line with other studies^11,29^, our results confirm that broadcasting preregistered videos is a valid methodology to investigate horses’ perception of humans. A two-dimensional image accompanied by sound is sufficient to make horses react behaviorally and physiologically, with reactions comparable to those observed in response to real-life corresponding stimuli (for example, in this study, the horses were more attentive during PDS, a behavior comparable to the higher number of looks toward the experimenter during grooming accompanied by PDS in our previous study^21^). Moreover, this method has the advantage of allowing the presentation to all the animals of the same standardized stimuli of humans who are blind to the reactions of the subject. In addition, compared to the broadcasting of sound alone, video projection enables the inclusion of facial expressions, an intrinsic component of PDS^16^.

The main result of this study is that horses were more attentive when addressed via PDS than ADS, which enables us to conclude that PDS can indeed attract horses’ attention. In addition, the heart rate of horses increased more during PDS than during ADS. In mammals, heart rate can fluctuate with arousal state (in both positive^33,34,36^ and negative^30,31,37^ situations), suggesting that horses in the present study were more aroused during PDS than ADS. Thus, PDS has an arousing effect on horses in addition to attracting their attention, as proposed in **H1**.

In the second hypothesis (**H2**, which does not exclude **H1**), we asked whether PDS influences the valence of emotions felt by horses. Our results show that the horses were not in a more positive or less negative emotional state during PDS than during ADS. The time spent in a relaxed posture was not longer during PDS than during ADS, and no snorts (an indicator of positive emotional state^32^) were observed during either PDS or ADS. Moreover, the horses were not less alert and did not defecate, neigh or show the sclera less during PDS than during ADS. Thus, we do not have sufficient evidence to accept **H2** and conclude that PDS influences the valence of emotions felt by horses and makes them more positive. However, in horses, contrary to other species such as dogs in which a high or wagging tail indicates confidence or friendliness^38^, few indicators of a positive emotional state, other than a relaxed posture, snorts, or a facial expression described during the specific context of grooming^26,29,32^, have been characterized, to our knowledge. Consequently, data are not in favor of **H2**; and more observable behavioral indicators of positive emotional states are needed to investigate this question further. Moreover, the tested horses expressed low levels of fear or stress, making it difficult to demonstrate a change toward a less negative emotional state due to a possible floor effect.

Several explanations can be proposed to interpret horses’ increased attention and arousal in response to PDS (**H1**). First, this effect could be due to the acoustic characteristics of PDS. Acoustic characteristics such as the mean pitch, pitch variation, pitch range and rate of speech, can be referred to as prosody, while the type of words used and their degree of repetition, along with syntax and semantics, can be referred to as content. In dogs, both prosody and content are important factors in the preference for PDS over ADS. Indeed, this preference is only revealed when the stimuli combine both factors as opposed to stimuli made either of PDS content spoken in ADS prosody or ADS content spoken in PDS prosody^17^. In our study, the same sentence was used for the PDS and ADS stimuli, so the content was stable but the prosody varied. It is possible that the differences in prosody, namely, a higher pitch and wider pitch range, could be more stimulating for horses than a more monotonous voice (ADS), similar to the suggested arousing effect of high pitch frequency and variation in infants^16^. In addition, in the present study, the attention of horses was drawn more efficiently by PDS, showing that in horses, for constant content, a different prosody is sufficient to promote greater attention (similar to the study by Jeannin et al.^19^ in dogs). It would be interesting to present horses with stimuli of stable prosody with a variation in content to determine if PDS content could also draw attention in this species.

Second, we can wonder whether the attention of horses was drawn more efficiently by PDS because this type of language enables them to better perceive our intention to communicate. Horses are known to perceive human intentions^13^. Indeed, in a protocol similar to those classically used in primates (“unwilling versus unable” paradigm)^39^, horses behaved differently toward an experimenter when the latter was able but unwilling to give a treat than when she was unable to do so (because the experimenter dropped the treat or a physical barrier prevented it)^13^. IDS is known to communicate parents’ intentions, and in infants, it is thought to work as an ostensive cue, alerting the child that communication is intended for them^16^. Dogs and cats have been shown to respond to ostensive cues as well^40–42^. Therefore, it is possible that horses could be sensitive to ostensive cues and that PDS could play this role. Hence, in this study, the greater attention and arousal of horses in response to PDS could be explained by improved perception of the filmed women’s intention to interact due to this type of speech.

A third possibility is that horses’ attention and arousal were increased in response to PDS due to the emotions conveyed by this type of speech. IDS is emotionally charged^16^, and in our study, the stimuli included emotional indicators such as facial expressions (for example, smiles during PDS and more neutral expressions during ADS). Horses are known to recognize positive human emotions, particularly joyful facial expressions and voices, in films^11^, and they react to face pictures of different human emotions^43^. Therefore, in this study, the perception of the positive emotional charge of PDS could have attracted the horses’ attention and aroused them.

Thus, the arousing and attention-attracting effect of PDS could be explained by the acoustic characteristics of PDS, better communication of intentions through this type of speech or attraction to the positive emotional charge of PDS. These three mechanisms could also be concomitant and function in a loop, with horses’ attention being drawn in response to acoustic stimulation, which would lead them to perceive our emotions and communicative intention and, in turn, increase their attention and arousal.

## Conclusion

In this study, we used an innovative setup in which horses watched and reacted to videos of women speaking in PDS or ADS. The results confirm horses’ sensitivity to PDS^21^ and provide some explanation of the mechanisms behind this phenomenon. We could not confirm that PDS influences the valence of emotions felt by horses, but it could be the case in other contexts (e.g., less pleasant situations). We showed that horses’ attention is drawn by PDS more than it is by ADS and that horses appear to be aroused by PDS. This finding could be helpful for horsemen and horsewomen, who could use this type of speech to attract horses’ attention and arouse them.

## Methods

### Ethics statement

Our experiment received a positive recommendation and was approved by the Val de Loire Ethical Committee (CEEA VdL, Nouzilly, France, authorization number CE19—2021-1101-1). Animal care and experimental treatments complied with the French and European guidelines for the housing and care of animals used for scientific purposes (European Union Directive 2010/63/EU) and were performed under authorization and supervision of official veterinary services (agreement number F371752 delivered to the UEPAO animal facility by the veterinary service of the Département d’Indre et Loire, France). The animals lived in groups, they were not food deprived during the experiment and did not undergo any invasive procedures. However, if a horse was too agitated during the test due to separation from its conspecifics or if it reacted too strongly to the videos (moved backward, turned around and tried to escape), it was removed from the experiment.

All methods were performed in accordance with relevant guidelines and regulations for direct human involvement in the study. All the people participating in the study provided their informed consent.

### Subjects

The study initially involved 32 Welsh mares aged 8.9 ± 2.4 years (mean ± sd) reared at the Animal Physiology Experimental Unit PAO (UEPAO, https://doi.org/10.15454/1.55738963217 28955E12), INRAE. The horses lived in groups in an indoor stall on straw with free access to an outdoor area and environmental enrichments. Fresh straw was added daily and the stalls were cleaned extensively every one to three weeks. The horses had free access to an outdoor paddock. Hay and water were available ad libitum. The whole protocol was successfully implemented with 28 of these animals, which were considered in the final statistical analysis (see “Habituation” and “Test” sections).

### Stimuli

The stimuli consisted of 2-min-long films composed of four 30-s sections. Each section consisted of one of four women speaking the same sentence four times in either PDS or ADS (Table 1). In each film, two women spoke in PDS and two others spoke in ADS. The order in which the types of speech appeared was counterbalanced between the films. The order in which the women appeared in the videos was counterbalanced between horses. The type of speech (ADS or PDS) used by each woman in front of the horses was also counterbalanced between horses (half of the horses saw two women speak in PDS and the other two women speak in ADS, and vice versa for the other half).

**Table 1:** Example composition of a stimulus film.

Section	1	2	3	4
Woman	A	B	C	D
Type of speech	ADS	PDS	ADS	PDS

A film lasted two minutes and was divided in four sections. During each section a same sentence was repeated four times. The orders of the types of speech, along with which type of speech each woman was using for each horse, were counterbalanced.

The films were recorded at the INRAE PRC lab. The four women were volunteers unknown to the horses. They were filmed saying the sentence « Oui mon beau, c’est bien tu écoutes très bien. Allez ma fille, tu viens, on y va, tu es la plus belle » (meaning « Yes my beauty, that’s good you’re listening very well. Come on girl, let’s go, you’re the most pretty »), made of phrases they were likely to hear in their everyday life. They were asked to speak it either with a neutral tone (ADS) or with a tone that they would use in front of a baby or cute juvenile animal (PDS), with the appropriate facial expressions for the type of speech used (smiling for PDS and neutral for ADS—see Supplementary Fig. S1). In the end, we obtained eight sequences of the same content that differed in prosody (four sequences of ADS and four of PDS). The sound amplitude was normalized so that the sound level would be between 65 et 75 dB from where the head of the horse stood for each sequence. The vocalizations were analyzed using Praat V6.1.16 (Supplementary Fig. S1). The mean pitch and pitch range were calculated for each woman. These analyses showed that the mean pitch was higher and the pitch range was wider in the PDS than in the ADS condition (mean pitch (Hz) PDS: 325[308;340], ADS: 239[217;254]; pitch range (Hz) PDS: 405[387;408], ADS: 168[129;211]). A high pitch and wide pitch range are two characteristics of PDS^16–21^.

### Experimental set-up

**Figure 2:**
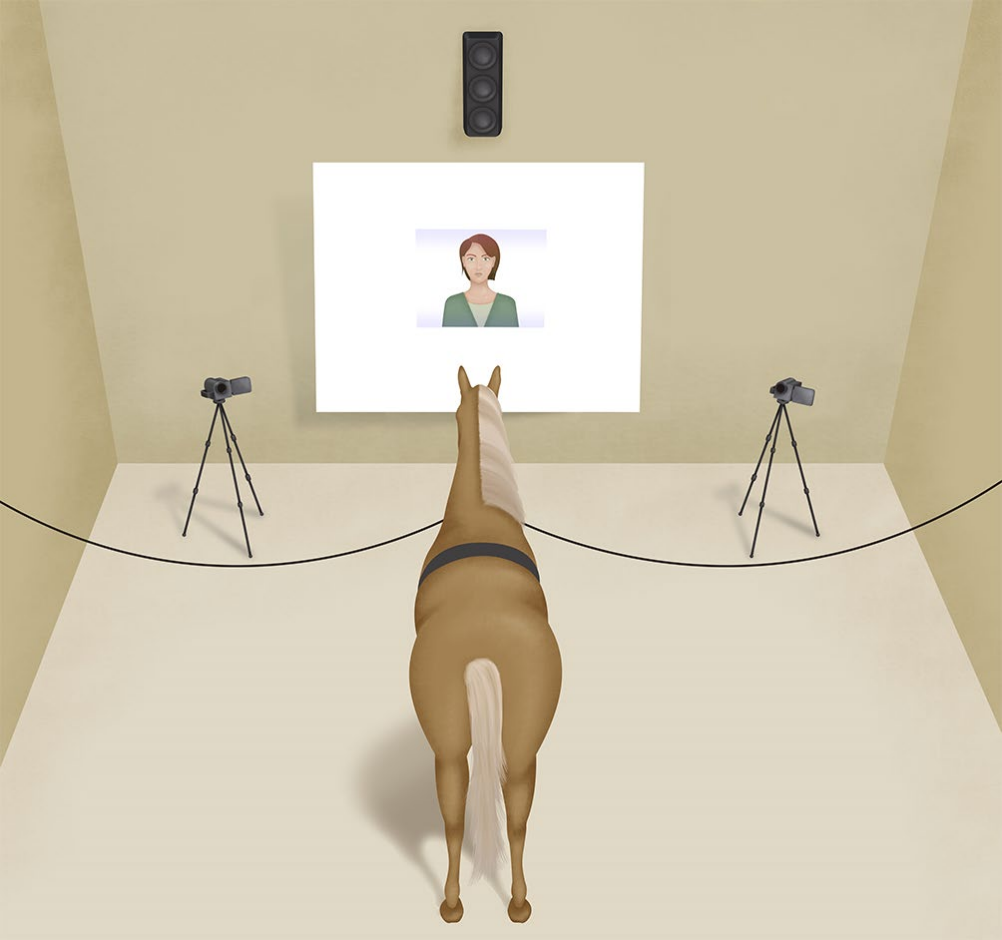
Schematic representation of the experimental setup.

The experiment was performed in a large stall (3.5 × 4.5 m). The films were projected on a white 2 × 2.5 m screen, so that the projected faces were approximately the size of a real person’s face (Fig. 2). The sound was broadcast by a speaker placed above the screen (approximately 70 dB, from where the head of the horse was located). For safety reasons, an assistant stayed with the horse (to ensure that it did not panic or get entangled in the ropes) but never interacted with the horse during the tests, remaining still and looking neither at the screen nor the horse, with the head down. The horses were free to look at the screen or not. Whether the assistant was standing on the left or the right of the horse was counterbalanced between the horses and the conditions. The experiment was filmed by two cameras in front of the horse (Fig. 2). An overview camera allowed the experimenter to follow the running of the experiment from outside the stall and control the projection accordingly. The horses were equipped with a heart monitor system composed of an external captor and a watch giving real-time values of heart rate and recording these values (Polar Equine RS800CX Science, Polar Oy, Finland). The external captor was composed of two electrodes placed on the withers and behind the front leg on the left, after clipping the hair and applying some ultrasound gel. The recordings started just before the start of the habituation phase, a signal was given to the camera at that moment to allow for synchronisation between the records and the proceedings of the test.

### Schedule

The experimental sessions were performed in the afternoon. On day 1, each horse went through a habituation session. If the habituation criterion was met (see below), the horse proceeded to the test session immediately on the same day; otherwise, a new habituation session with the same criterion was scheduled for the following day. All but one horses met the criterion on day 1 and proceeded to the test session on the same day. One horse did not reach the criterion on day 1 or day 2 and was excluded from the experiment.

### Habituation

The horse was led to the middle of the stall facing the screen. Two loose ropes were attached, the assistant took his place, and the habituation phase began. Scenes of nature accompanied by bird songs were projected while the assistant monitored the horse’s heart rate on the Polar watch. In this phase, the assistant could reposition the horse if it was facing the door opposite the screen. Once the horse was calm (not neighing, pulling on the ropes with his head, nor trying to turn around or leave) and its heart rate had remained below 100 bpm for two consecutive minutes, the test phase began immediately. If this criterion was not met after five minutes, the session ended, and a new session was scheduled for the following day.

### Test

Immediately after the horse met the criterion, a film presenting the four women speaking in PDS and ADS (see “Stimuli” section above) was projected. The conditions were the same as during habituation, but the assistant did not intervene, unless the horse’s level of stress was too high. This occurred three times, and the horses in question had to be excluded from the study (one horse behaved dangerously and two were agitated and tried to escape). At the end of the test, the horse was led directly back to its stall.

### Behavioral and physiological analysis

Videos of the tests were watched on a standard media player (http://www.videolan.org/) by the same coder without sound, so that the coder was blind to the condition (the screen was not visible in the videos). The start and end times or times of occurrence of the behaviors were noted. The time the horse spent being attentive to the film (facing the screen with both ears oriented forward) was quantified. Signs of negative emotions were also recorded: defecations, the number of times the white of an eye was visible (shows of sclera), and neighs (based on a second viewing with the sound on) were counted, and the time the horse spent in an alert posture (neck held high with both ears oriented forward, regardless of the gaze direction) was quantified. Signs of positive emotions were also recorded: the time the horse spent in a relaxed posture (neck held low or medium, eyes weakly or moderately open, regardless of the gaze direction) was quantified, and the number of snorts (defined as a voluntary expulsion of air through the nostrils that make them vibrate and results in a pulsed sound^32^) was counted. A second coder reanalyzed 20% of the videos to assess the interobserver reliability of the times spent being attentive to the video, in a relaxed posture or in an alert posture. Interclass Correlation Coefficients (ICC) were calculated, showing good to excellent reliabilities for the three variables^44^ (ICC estimates and 95% confidence intervals: time spent being attentive to the screen 0.93[0.85,0.97], time spent in a relaxed posture 0.93[0.84,0.97], time spent in an alert posture 0.94[0.87,0.97]).

The time spent being attentive to the screen was highly variable between horses (from 6 to 80 s over the 120-s test). To take this variation into account, for each horse, we calculated an attention index measuring the propensity to be more attentive to PDS than ADS relative to the total time spent being attentive to the screen. This index was defined as (A^PDS^-A^ADS^)/(A^PDS^ + A^ADS^), where A^PDS^ is the time spent being attentive to the screen during PDS sections and A^ADS^ is the time spent being attentive during ADS sections (see^4^). This index varied from -1 to 1, with a negative value indicating that a horse was more attentive during ADS than PDS, and a positive value indicating the opposite.

Heart rate data were extracted from the Polar recordings. A visual correction was applied to eliminate artifactual beats (as recommended in^45^). The difference in heart rate (beats per minute—bpm) between the last five and first five seconds of each section was calculated. Then, for each horse, the mean of this difference over the two sections of each type (PDS or ADS) was calculated.

### Statistical analyses

Due to the exclusion of one horse during habituation and three horses during the tests, the statistical analysis considered 28 animals. All statistical analyses were performed using R 4.0.3 (R Core Team, 2013). Due to the small sample size (N = 28), nonparametric tests were used. The significance threshold was fixed at α = 0.05.

To test whether the attention index was greater than 0 (**H1**), which would indicate that the horses spent a longer period of time being attentive to the screen during PDS than during ADS sections, we used a one-tailed paired Wilcoxon test. To test whether the horses spent more time in a relaxed posture and less time in an alert posture during PDS than during ADS (**H2**), we used one-tailed paired Wilcoxon tests. Defecations, neighs and shows of the sclera were expressed by less than 30% of the horses, so the corresponding observations were transformed into binary data. We used one-tailed Z tests to determine whether the proportions of horses that expressed each of these behaviors during PDS were smaller than those during ADS (**H2**). Finally, we tested whether the mean difference in heart rate over PDS sections was greater (**H1**) or smaller (**H2**) than that over ADS sections using a two-tailed paired Wilcoxon test, and we tested whether the heart rate variation of horses over each type of section was different from 0 using two two-tailed Wilcoxon tests.

### Ethical approval

This study was reported in accordance with ARRIVE guidelines.

## Supplementary Information

Supplementary Figure S1.

## Data Availability

The datasets generated and analyzed during the current study are available in the INRAE data repository from the following link: https://doi.org/10.15454/T9DW8Y.
