# Supplementary material for: Pet-directed speech improves horses’ attention toward humans
Source: Sci Rep. 2022 Mar 11;12:4297. doi: 10.1038/s41598-022-08109-z (PMC8917202; doi:10.1038/s41598-022-08109-z)
Supplement: Supplementary file 1 — Supplementary Figure S1. [file 41598_2022_8109_MOESM1_ESM.docx]

Pet-directed speech improves horses’ attention toward humans

Plotine Jardat^1*^, Ludovic Calandreau^1^, Vitor Ferreira^1,2^, Chloé Gouyet^1^, Céline Parias^1^, Fabrice Reigner^3^, Léa Lansade^1^*

Affiliations:

^1^CNRS, IFCE, INRAE, University of Tours, PRC, 37380 Nouzilly, France

^2^Department of Physics, Chemistry and Biology, IFM Biology, Linköping University, 581 83 15 Linköping, Sweden

^3^UEPAO, INRAE, F-37380, Nouzilly, France

*corresponding authors
ORCID IDs: P Jardat 0000-0003-0374-5588 – mail: [plotine.jardat@gmail.com](mailto:plotine.jardat@gmail.com)

L Lansade 0000-0002-4185-9714 – mail: [lea.lansade@inrae.fr](mailto:lea.lansade@inrae.fr)


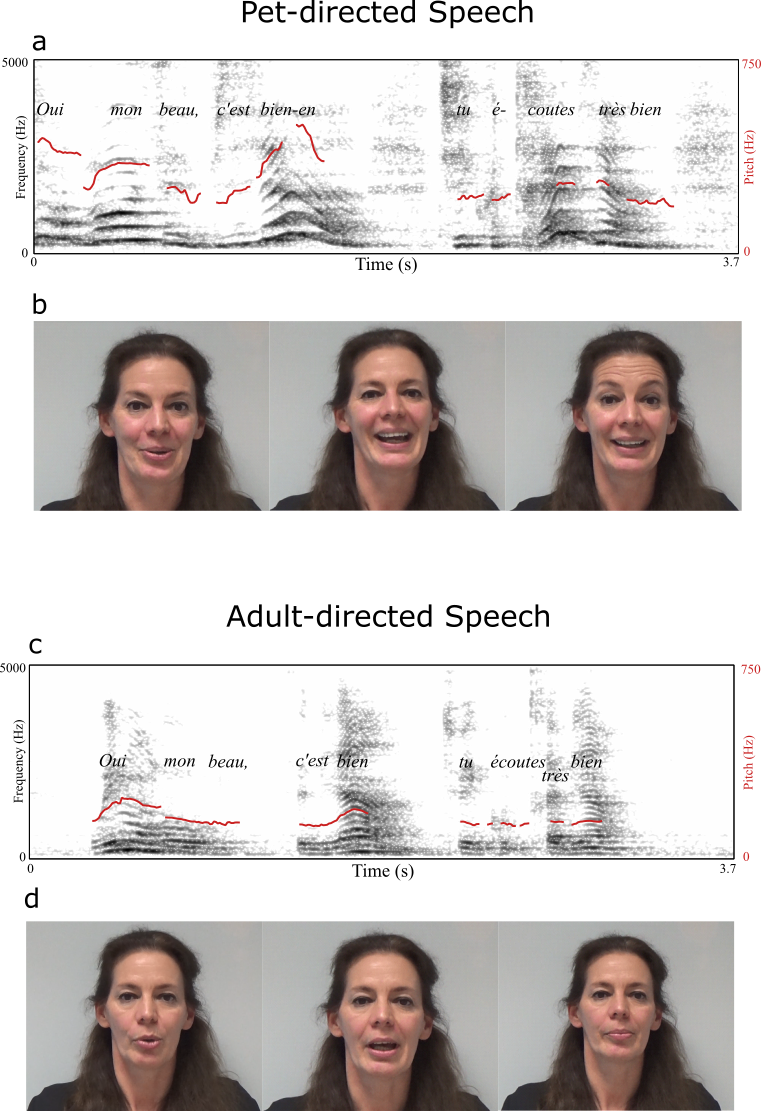


Figure S1: Example spectrograms and screenshots of the stimuli. **a, b**: Pet-directed speech; **c, d**: Adult-directed speech. The spectrograms and pitch were produced using Praat Spectrogram and Pitch functions respectively (https://praat.fr.softonic.com/). Spectrogram settings: Fourier method, window length 0.03 s, number of time steps: 1000 and frequency steps: 250, Gaussian window, dynamic range: 45 dB.
